# Relationship between miR-378c and YY1 expression in patients with gastric cancer and the clinicopathological features

**DOI:** 10.1186/s11658-021-00256-x

**Published:** 2021-04-01

**Authors:** Lei Zhang, Lei Zou, Peng Sun

**Affiliations:** Department of Gastroenterology, Zibo Central Hospital, No. 54 Gongqingtuan West Road, Zhangdian District, Zibo, 255000 Shandong China

**Keywords:** miR-378c, YY1, Gastric cancer, Prognosis

## Abstract

**Background:**

The purpose of this study was to explore the clinical value of miR-378c and its target gene YY1 in gastric cancer.

**Methods:**

The TCGA database was employed to analyse miR-378c expression in gastric cancer. qRT-PCR was applied to identify miR-378c and YY1 in tissues and serum of patients suffering from gastric cancer. The association of miR-378c with the clinical data of patients with gastric cancer was analysed. Receiver operating characteristics (ROC) curve analysis was used to determine the diagnostic value of miR-378c and YY1 in gastric cancer, and analyse the relationship between miR-378c and YY1 and patients’ survival. Pearson’s test was applied to determine the association between miR-378c and YY1 in tissue and serum of patients. Dual-Luciferase Reporter assay was employed to examine the targeting association between miR-378c and YY1. Finally, independent prognostic factors was determined in patients with gastric cancer using Cox regression analysis.

**Results:**

In the TCGA database, miR-378c was weakly expressed in gastric cancer. Overall, patients with low expression had a lower survival rate. The expression of miR-378c decreased and the expression of YY1 increased in cancer tissues and serum of tumour patients. In patients with low expression of miR-378c the tumour size was ≥ 5 cm. Low differentiation, high TNM staging and lymph node invasion rate increased significantly, but the 5-year survival rate decreased in the patients. miR-378c and YY1 had better diagnostic value in gastric cancer. TargetScan, miRDB, starBase and miRTarBase predicted that YY1 was a potential gene of miR-378c, and the Dual-Luciferase Reporter assay revealed that there was a targeting relationship between the two, which was proved by correlation analysis. Multivariate Cox analysis revealed that differentiation, TNM staging and miR-378c were independent prognostic factors for patients.

**Conclusions:**

MiR-378c is weakly expressed in gastric cancer patients and may be considered as a promising diagnostic and prognostic indicator for gastric cancer.

## Background

Gastric cancer is a clinically common malignant tumour. It is the third leading cause of death from malignant tumours worldwide [1]. A review of the recent global tumour statistics report [2] indicated that more than 1 million new cases and 7,00,000 deaths from gastric cancer were registered in 2018. It has also been found that the onset age of gastric cancer in patients is gradually getting younger. Therefore, the prevention of gastric cancer is one of the urgent problems for clinicians to solve. Clinically, gastric cancer is treated mainly through surgery. However, due to the lack of typical early symptoms, most patients with gastric cancer are diagnosed at an advanced stage, and it is difficult for patients with advanced stage gastric cancer to undergo surgical treatment, resulting in a poor prognosis [3, 4] Although researchers have explored the development of gastric cancer and related molecular mechanisms in recent years [5], it is still a great challenge to find new targets for diagnosis and treatment of gastric cancer.

Recently, an increasing number of studies have revealed that non-coding RNA is closely related to the development of tumours [6], in which microRNA (miR) has a key role in the prevalence of tumours. miR is an endogenous short-chain non-coding RNA having an approximate length of 17–25 nt, and it has a targeted regulation effect [7, 8]. Some studies have revealed that miR can degrade target gene mRNA or inhibit mRNA translation through sequence-specific interaction at the 3ʹ-UTR end [9]. Recently, an increasing number of studies have revealed that miR is closely related to the prognosis of gastric cancer. For example, An et al. [10] revealed in their study that miR-1236-3p is a potential new biomarker that can be used for diagnosis and prognosis of gastric cancer. Other studies [11] have further revealed its application in diagnosis and prognosis of gastric cancer by detecting miR-25 expression in serum of patients with gastric cancer. In the present study, the analysis of The Cancer Genome Atlas (TCGA) database [12] indicated that miR-378c was weakly expressed in gastric cancer patients, and the difference was highly significant. However, there are relatively few studies on its role in gastric cancer.

Yin Yang 1 (YY1) is a transcription factor belonging to the GLI-Krüppel class of proteins, plays essential roles in a multitude of biological processes, and has important roles in carcinogenesis [13, 14].

Therefore, this study aimed to provide a potential therapeutic target for the clinic by exploring the clinical value of miR-378c in gastric cancer and its downstream target genes.

## Materials and methods

### Clinical data

From January 2013 to September 2014, 122 patients with gastric cancer who were treated in our hospital were collected and included in the patient group (PG) in this study. Another 50 normal people who underwent physical examination in our hospital were collected and included in the control group (CG) in this study. There was no statistically significant difference in gender or age between the two groups. Before treatment, the peripheral blood was drawn and centrifuged from patients in the CG and PG, and the supernatant was collected and stored at − 80 ℃ for testing. During the operation, tissues infected with gastric cancer and the adjacent tissues were collected from patients, conveyed in liquid nitrogen and finally maintained at − 80 ℃ for testing.

Inclusion criteria for patients: The patient was diagnosed with gastric adenocarcinoma through pathological examination, and the patient met the 8th edition of TNM staging criteria [15]. The patient received no treatment for his/her tumour before the study. The patient was informed of the purpose of this study and filled out a consent form.

Exclusion criteria in this study were patients having other tumour types, patients who were unable to cooperate with follow-up, and patients were expected to survive for less than 1 month.

### TCGA database analysis

We logged in to https://portal.gdc.cancer.gov/, downloaded quantitative miRNA expression data of gastric adenocarcinoma transcripts, and synthesized matrix files. The edgeR package was used to analyse the differences of matrix files.

Clinical data of gastric adenocarcinoma patients were obtained from http://gdac.broadinstitute.org/runs/stddata__2016_01_28/data/STAD/20160128/ in order to analyse the association of miR-378c with the survival rate in patients with gastric adenocarcinoma.

### Quantitative reverse transcription PCR (qRT-PCR) and RNA extraction

A TRIzol kit (Invitrogen Company, USA) was used to extract total RNA. Agarose gel electrophoresis and UV spectrophotometry were used to detect the concentration, integrity and purity of total RNA. Subsequently, to perform reverse transcription, a TaqMan Reverse transcription kit (Invitrogen, USA) was used and operated according to the kit instructions. The obtained cDNA was considered for further study. A PrimeScript RT Master Mix kit (Takarabo Company, Japan) was applied to amplify the PCR. The kit instructions were followed carefully when amplifying the system reaction. In this study, 3 parallel multiple pores were designed, and the experiments were performed on specimens in triplicate. In the experiments, GAPDH and U6 were used as an internal reference in GENE and miR, respectively. 2^−△△ct^ was used to analyse the data [16]. 7500PCR was the PCR instrument in ABI.

### Follow‐up of patients

Patients were followed up for survival for 5 years. All patients were counted by telephone and outpatient electronic pathology. The follow-up times in each year were the five months of January, March, June, September and December.

### Prediction and identification of target genes

In the present study, the target gene of miR-378c was predicted using online databases TargetScan [17], miRDB [18], starBase [19], and miRTarBase [20], and the weien picture was drawn by bioinformatics to search for common predictive target genes. In addition, the miR-378c mimetic (miR-378c mimics) and control (miR-NC) were synthesized into TM by GeneCoposia (Guangzhou, China). Partial sequences of YY1-3ʹUTR containing predetermined miR-378c binding sites were amplified by PCR and introduced to the pmiRGLO report vector (Promega Corp., Madison, WI, USA). Mutant (YY1-, MUT) and wild-type (YY1-WT) gene reports were constructed. The human renal epithelial cell line (293t) cells were co-transfected with the constructed gene report and miR-NC by Lipofectamine 3000 reagent. The relative activity of luciferase was analysed after transfection for 48 h using a Dual-Luciferase Reporter assay kit (Promega).

#### Statistical analysis

The pictures required for this study were drawn using GraphPad 7. Independent prognostic factors of patients were analysed in SPSS 20.0. The independent sample T test and paired T test were used for inter-group and intra-group comparison, respectively. The numeric data were expressed as a percentage (%). Gene correlation analysis was performed using Pearson’ test. ROC was used to plot the diagnostic value of miR-378c in gastric cancer. Total survival of patients was plotted by a Kaplan-Meier survival curve and analysed using the log-rank test. Multivariate Cox regression was employed to analyse the prognosis of patients. The chi-square test was represented by χ^2^. A P-value lower than 0.05 was considered as statistically significant.

## Results

### Expression and clinical value of miR-378c in gastric cancer

**Fig. 1:**
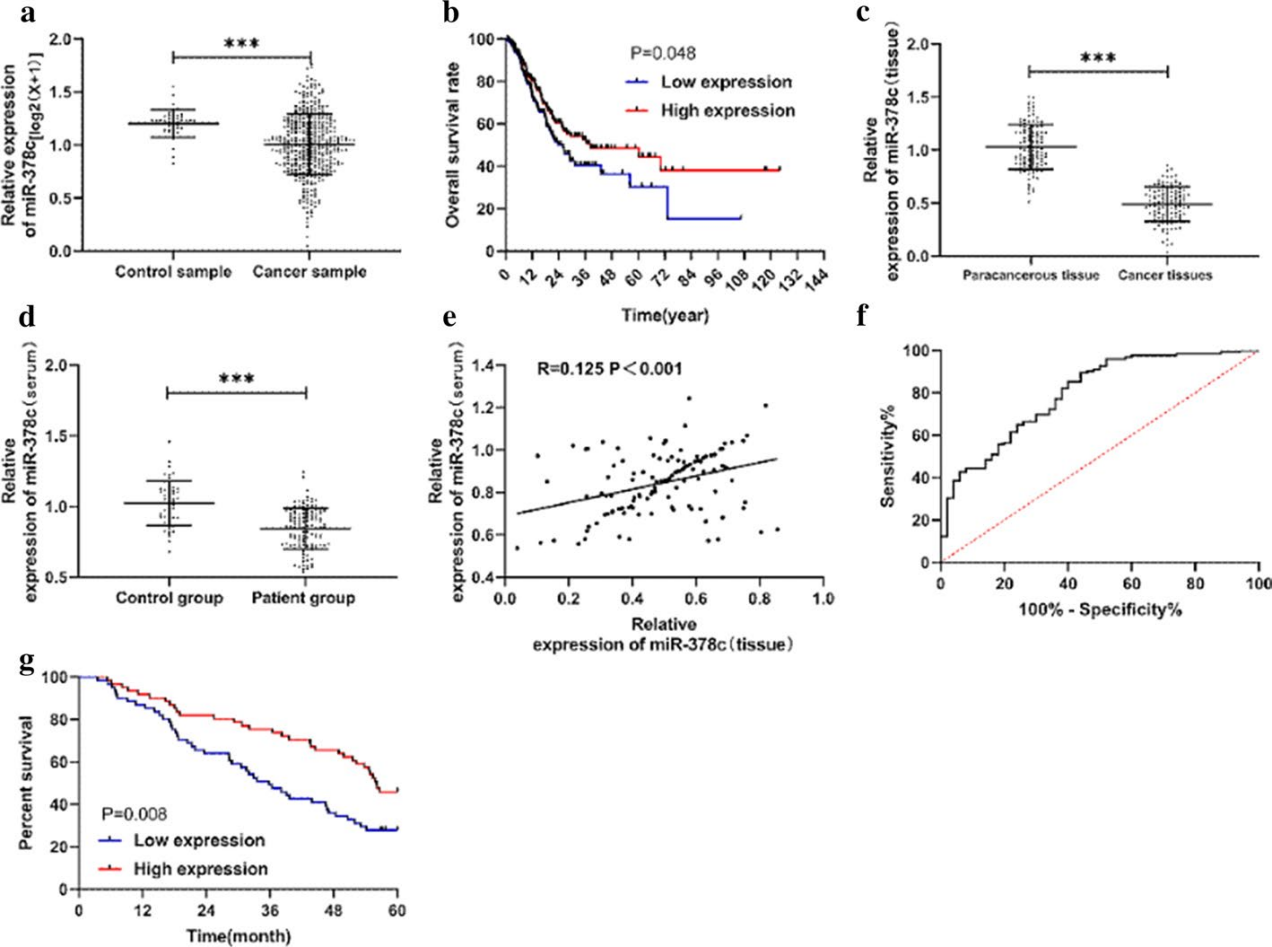
Expression and clinical value of miR-378c in gastric cancer. **a** Relative expression of miR-378c in gastric cancer in TCGA database. **b** Relationship between miR-378c in TCGA database and the total survival of patients with gastric cancer. **c** Expression of miR-378c in the tissues of patients with gastric cancer. **d** Expression of miR-378c in serum of patients with gastric cancer. **e** Correlation analysis of miR-378c in serum and tumour tissue of patients with gastric cancer. **f** ROC curve of miR-378c expression in serum in the diagnosis of gastric cancer. **g** Relationship between miR-378c and survival of clinical patients. *** P < 0.001. R = r^2^

The analysis of miR-378c expression in the TCGA database indicated weak expression of miR-378c in gastric cancer (Fig. 1a). The association of miR-378c and survival was also analysed in this study and the results showed that patients with lower miR-378c expression had a lower survival rate (Fig. 1b). In order to verify the above conclusions, we collected clinical samples and detected the expression of miR-378c in tumour tissue and serum of patients. miR-378c expression was found to be lower in tumour tissue and serum of patients with tumours (Fig. 1c, d). Correlation analysis showed that the miR-378c expression in cancer tissue and serum was positively correlated (Fig. 1e). The ROC curve analysis indicated that the area of miR-378c in serum under the curve in diagnosing gastric cancer was 0.798, which had better clinical value (Fig. 1f). Moreover, the correlation analysis of miR-378c and patients’ clinical data indicated that patients with low expression had tumours ≥ 5 cm, and low differentiation, high TNM staging and lymph node invasion rate increased significantly (Table 1). In addition, patients with higher miR-378c expression showed a lower survival rate after a 5-year follow-up period (Fig. 1g).

**Table 1:** Relationship between miR-378c and pathological data of patients with gastric cancer

Parameters	Relative expression of miR-378c		P
	High expression group (n = 61)	Low expression group (n = 61)	
Age			
≥ 60 years old (n = 53)	25	28	0.584
< 60 years old (n = 69)	36	33	
Gender			
Male (n = 80)	37	43	0.253
Female (n = 42)	24	18	
Tumor size			
≥ 5 cm (n = 38)	12	26	0.006
< 5 cm (n = 84)	49	35	
Differentiation			
Low differentiation (n = 52)	17	35	0.001
Middle + High differentiation (n = 70)	44	26	
Lymph node invasion			
Yes (n = 49)	17	32	0.006
No (n = 73)	44	29	
TNM staging			
Stage I + II (n = 64)	40	24	0.004
Stage III + IV (n = 58)	21	37	

## Prediction of miR-378c target gene

**Fig. 2:**
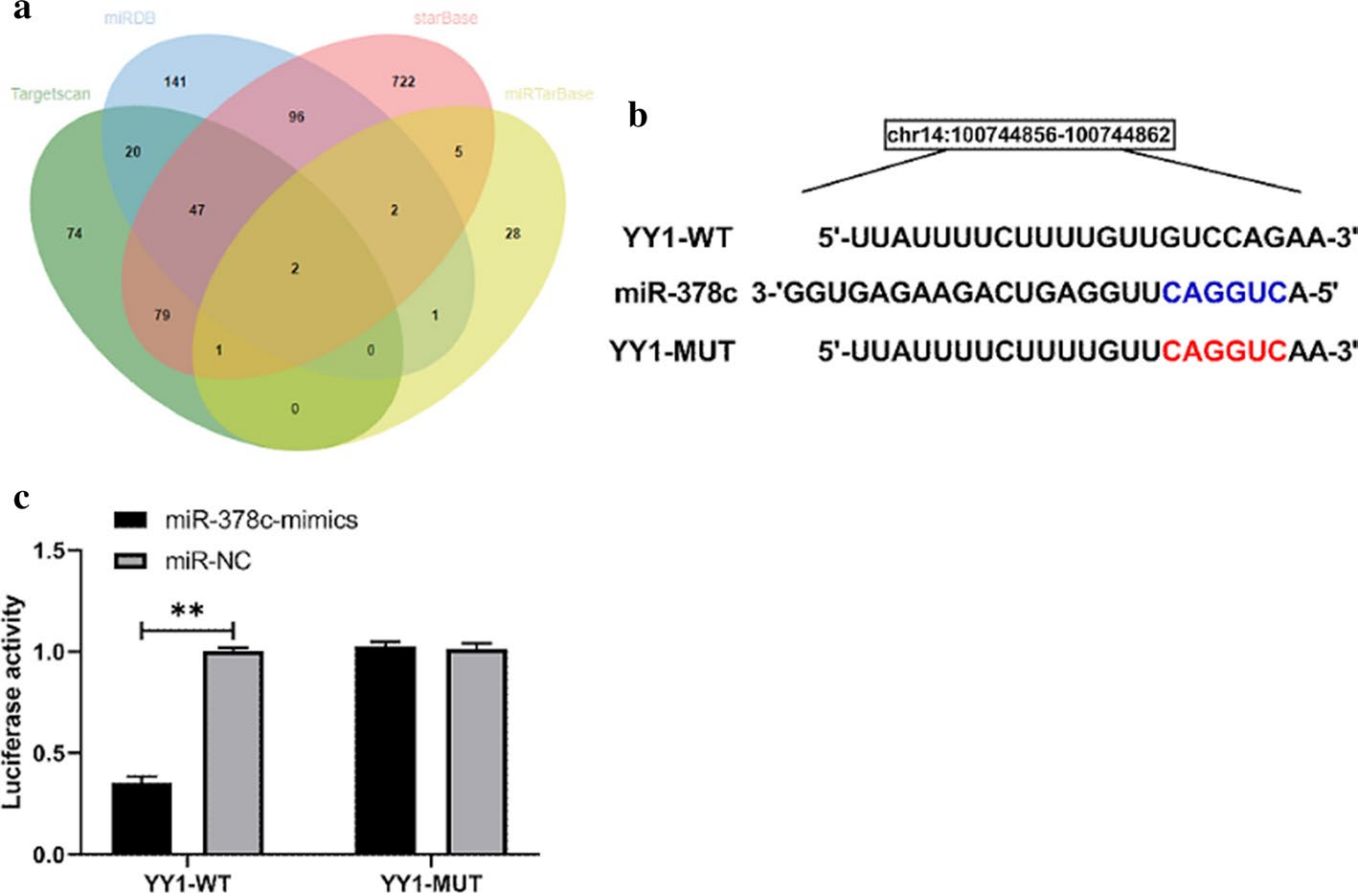
Prediction and identification of miR-378c target gene. **a** TargetScan, miRDB, starBase, miRTarBase online miR prediction websites were used to predict miR-378c target genes. **b** Binding and mutation sites of miR-378c and YY1. **c** Dual-Luciferase Reporter assay revealed that miR-378c had a targeting relationship with ROCK1. **P<0.01

Online prediction websites including TargetScan, miRTarBase, starBase, and miRDB were used to predict common miR target genes, and the result was 2 potential targets, including YY1 (Fig. 2a). YY1 has been shown to be weakly expressed in patients with gastric cancer. In this study, Dual-Luciferase Reporter analysis was conducted to verify the association of YY1 and miR-378c (Fig. 2b). The results indicated that fluorescence activity of YY1-WT can be inhibited by miR-378c mimics, which indicated that YY1 was a downstream target gene of miR-378c (Fig. 2c).

### Expression and clinical value of YY1 in gastric cancer

**Fig. 3:**
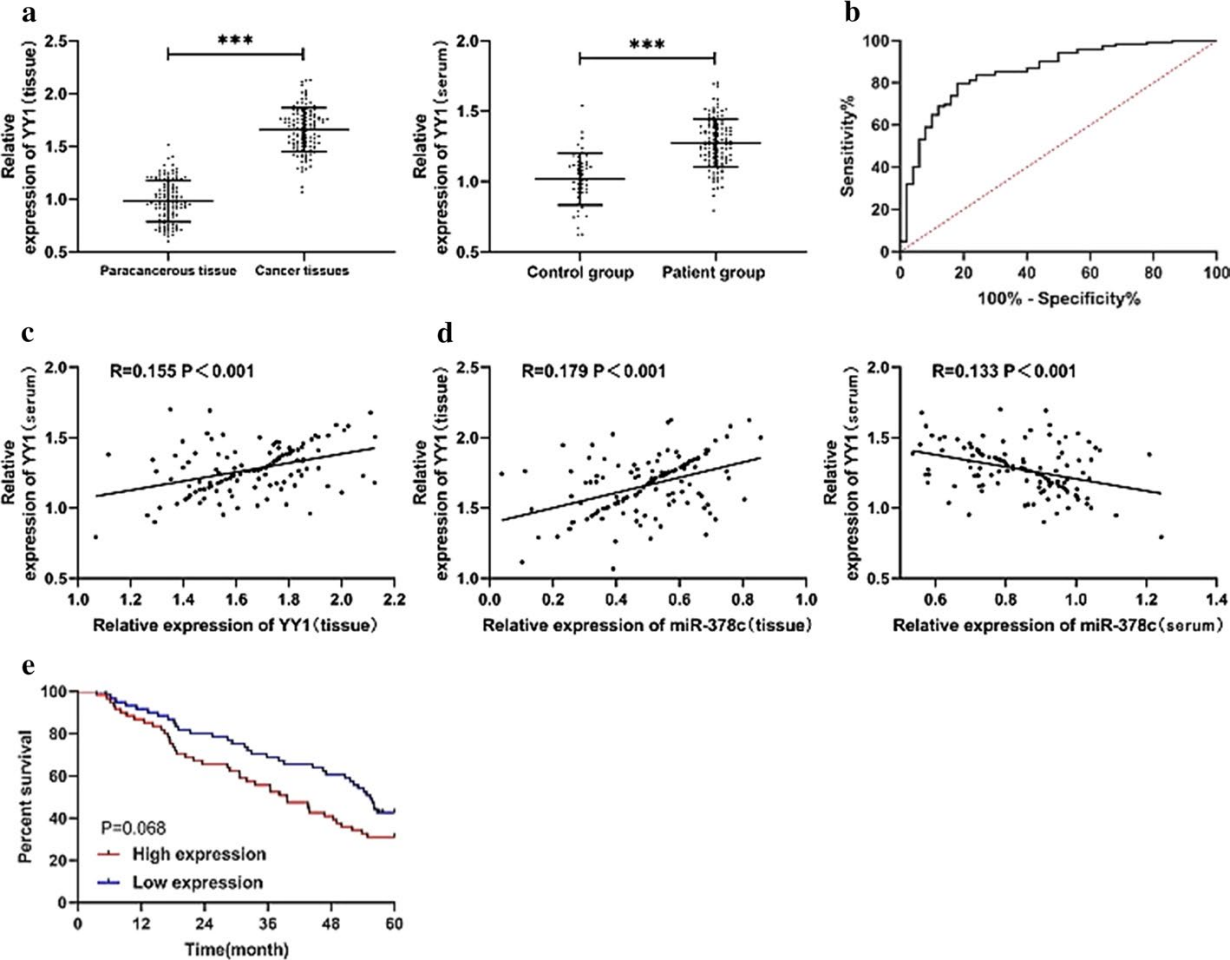
Expression of YY1 in patients with gastric cancer. **a** Relative expression of YY1 in gastric cancer tissues and serum. **b** ROC curve of YY1 in diagnosis of gastric cancer. **c** Correlation analysis of YY1 in tissues and serum of patients with gastric cancer. **d** Correlation analysis of relative expression of miR-378c and YY1 in cancer tissue and serum of patients with gastric cancer. E: Relationship between YY1 and survival of patients with gastric cancer. ***P < 0.001. R = r^2^

The above studies revealed the targeting association between YY1 and miR-378c. To determine the clinical value of YY1 in gastric cancer, we examined the expression of YY1 in tissues and serum of patients with gastric cancer. The results showed that YY1 expression in tissues and serum of patients was significantly increased (Fig. 3a). The correlation analysis revealed that YY1 expression in tissues and serum was positively correlated (Fig. 3c). ROC curve analysis showed that the curve area of YY1 in the diagnosis of gastric cancer was 0.856, which was of high clinical diagnostic value (Fig. 3b). However, we found that there was no correlation between YY1 and patients’ survival through correlation analysis (Fig. 3e). Correlation analysis revealed that the expression of YY1 in patients’ tissue and serum was negatively correlated with miR-378c (Fig. 3d).

### Cox regression analysis

At the end of the study, we carried out Cox regression analysis on the factors affecting the prognosis of gastric cancer. The results showed that differentiation, lymph node invasion, TNM stage and miR378c were the factors affecting the prognosis of patients through univariate Cox regression analysis. Furthermore, the indicators with significance as single factors were analysed using backward logistic regression for multi-factor analysis. The results showed that differentiation, TNM stage and miR378c were the independent prognostic factors of patients (Table 2).

**Table 2:** Prognostic factors of gastric cancer

Factors	Univariate Cox regression			Multivariate Cox regression		
	P	HR	95 %CI	P	HR	95 %CI
Age	0.748	0.928	0.590–1.461			
Gender	0.284	0.776	0.487–1.234			
Tumor size	0.120	1.469	0.905–2.386			
Differentiation	0.000	3.539	2.220–5.642	0.011	1.957	1.166–3.285
Lymph node invasion	0.000	2.773	1.756–4.376	0.728	1.101	0.641–1.888
TNM staging	0.000	0.287	0.179–0.460	0.500	0.297	0.297–0.842
miR378c	0.000	4.793	2.970–7.735	2.545	1.451	1.451–4.462
YY1	0.070	1.515	0.966–2.376			

## Discussion

As a common malignant digestive system tumour, gastric cancer is an important cause of cancer mortality worldwide [21]. However, up to now, the treatment and prognosis of patients with gastric cancer have not made ideal progress [18]. One of the main reasons is that the pathogenesis of gastric cancer is still unclear and there is a lack of biomarkers for early diagnosis of gastric cancer [22]. Therefore, it is necessary to further clarify the relevant mechanism of gastric cancer and find potential diagnostic biomarkers for gastric cancer to treat patients and improve prognosis.

miR is a kind of conservative short-chain non-coding RNA having an approximate length of 21–25 nt. Some research has demonstrated that miRs participate in various biological pathways of the body, and they can complement each other by targeting the 3ʹ untranslated region (3ʹUTR) of the target gene, which plays a role in regulating transcription of the target gene and reducing the expression of mRNA [23–25]. In recent years, an increasing number of studies have revealed that miR has high clinical value in diagnosing tumours. For example, Chen et al. [25] demonstrated that miR-296-5p inhibits cell metastasis and invasion-mesenchymal transition of nasopharyngeal carcinoma by reversing epithelial cells induced by transforming growth factor-β. Additionally, Tian et al. [26] demonstrated that miR-125b plays an anti-tumour role in cutaneous squamous cell carcinoma by targeting the STAT3 pathway. As a member of the miR family, miR-378c is situated on human chromosome 10q26.3. The expression of miR-378c in tumours has not been reported in previous studies. Only one study [27] has shown that the decreased expression of miR − 378 is related to tumour invasion and poor prognosis of patients with glioma. In this study, TCGA database analysis was conducted to find potential diagnostic markers for gastric cancer, and the results indicated weak expression of miR-378c in patients with gastric cancer. Moreover, the results of survival analysis showed that patients with low expression of miR-378c had significantly lower survival rates. This suggested that miR-378c might be a potential diagnostic and prognostic indicator for gastric cancer. In parallel, Liu et al. [28], revealed that serum miR-378 may be utilized as a noninvasive biomarker with strong potential in the diagnosis of gastric cancer, and another study by Gungormez et al. [29] showed that miR-378 was also weakly expressed in colorectal cancer, suggesting that miR-378 is also differentially expressed in other tumours. We have also speculated that miR-378 might have a potential regulatory role in gastrointestinal tumours.

To verify the clinical value of miR-378c in gastric carcinoma, samples of clinical patients were collected for detection. The results of the experiments showed that the expression of miR-378c was low in cancer tissues and serum of patients. The correlation analysis indicated that miR-378c expression in patients’ tissues and serum was positively associated. The use of miR expression as a diagnostic indicator for tumours has been suggested in several studies [30]. ROC curves were drawn to verify the diagnostic value of miR-378c in gastric cancer, and the results showed that the area under the curve of serum miR-378c in diagnosing gastric cancer was more than 0.7, revealing it to be an ideal biomarker. In addition, the 5-year follow-up results showed a decreased survival rate in patients with lower miR-378c expression. All the above conclusions indicated that miR-378c could be used as a potential diagnostic and prognostic marker for gastric cancer. However, its relevant mechanism is not clear. In order to pave the way for future research, we predicted the downstream target genes of miR-378c using online prediction websites, including TargetScan, miRTarBase, starBase and miRDB. Through joint prediction, we found that YY1 and miR-378c had binding sites. As a GLI-Kruppel class of zinc finger protein, YY1 is a well-distributed transcription factor [31]. Studies have revealed that [32] YY1 is highly expressed in gastric cancer. The Dual-Luciferase Reporter assay revealed that YY1-WT fluorescence activity could be inhibited by miR-378c mimics, verifying that YY1 is a potential downstream target gene of miR-378c. In addition, Wang et al. reported that the decrease of H2B monoubiquitination (uH2B) may contribute to tumourigenesis and could be a potential therapeutic target in the gastric tissues [33]. And another study by Kang et al. revealed that YY1 contributes to gastric carcinogenesis in gastric cancer through promoting cell survival in GAC cells and overexpression of YY1 enhanced cell proliferation by activating the Wnt/β-catenin signalling pathway [14].

In addition, in order to determine YY1 value in gastric cancer, the experiments showed that YY1 expression in tissues and serum of patients with gastric cancer was obviously increased, and the expression of serum and tissues was positively correlated. ROC curve analysis showed that the area under the curve of YY1 exceeded 0.8 in the diagnosis of gastric cancer, which was an ideal diagnostic index. However, survival analysis showed no statistically significant association between YY1 and 5-year survival of patients.

## Conclusions

In summary, miR378c expression is low in patients with gastric cancer, indicating its potential to be used as a diagnostic and prognostic indicator for gastric cancer. At the end of the study, we conducted a Cox regression analysis on the factors affecting patients with gastric cancer. Multivariate analysis revealed that differentiation, TNM staging and miR378c were effective, independent factors in the prognosis of patients. Differentiation and TNM staging have been previously reported as independent factors in the prognosis of gastric tumour, but this is the first time that miR378c has been detected as an independent prognostic factor of gastric cancer. However, there are still certain limitations in our research. As a clinical validation, the relevant mechanism of miR378c has not been further explored in this study. We will conduct in vivo and in vitro experiments in future studies to further improve the mechanism of miR-378c and YY1 and supplement our conclusions.

## Data Availability

The datasets used and/or analysed during the present study are available from the corresponding author on reasonable request.
